# Navigating the Crossroads: Understanding the Link Between Chronic Kidney Disease and Cardiovascular Health

**DOI:** 10.7759/cureus.51362

**Published:** 2023-12-30

**Authors:** Danish Saeed, Taufiqa Reza, Muhammad Waqas Shahzad, Alishba Karim Mandokhail, Danyal Bakht, Farwa Haider Qizilbash, Elizabeth O Silloca-Cabana, Afif Ramadhan, Syed Faqeer Hussain Bokhari

**Affiliations:** 1 Internal Medicine, Shaikh Zayed Medical Complex, Lahore, PAK; 2 Internal Medicine, Avalon University School of Medicine, Youngstown, USA; 3 General Practice, Gujranwala Medical College, Gujranwala, PAK; 4 Radiology, Quetta Institute of Medical Sciences, Quetta, PAK; 5 Medicine and Surgery, Mayo Hospital, Lahore, PAK; 6 Internal Medicine, Dow Medical College, Karachi, PAK; 7 Internal Medicine, Wyckoff Heights Medical Center, Brooklyn, USA; 8 General Practice, Faculty of Medicine, Public Health, and Nursing, Gadjah Mada University, Yogyakarta, IDN; 9 Surgery, King Edward Medical University, Lahore, PAK

**Keywords:** risk assessment, mortality, cardiovascular events, gfr, glomerular filtration rate, kidney damage, cvd, cardiovascular disease, ckd, chronic kidney disease

## Abstract

Chronic Kidney Disease (CKD) has emerged as a global healthcare challenge affecting a significant portion of the world's population. This comprehensive narrative review delves into the intricate relationship between CKD and cardiovascular disease (CVD). CKD is characterized by kidney damage persisting for at least three months, often with or without a decline in glomerular filtration rate (GFR). It is closely linked with CVD, as individuals with CKD face a high risk of cardiovascular events, making cardiovascular-associated mortality a significant concern in advanced CKD stages. The review emphasizes the importance of precise risk assessment using biomarkers, advanced imaging, and tailored medication strategies to mitigate cardiovascular risks in CKD patients. Lifestyle modifications, early intervention, and patient-centered care are crucial in managing both conditions. Challenges in awareness and recognition of CKD and the need for comprehensive interdisciplinary care are highlighted. Recent advances in research offer promising therapies, such as SGLT2 inhibitors, MRAs, GLP-1R agonists, and selective endothelin receptor antagonists. Stem cell-based therapies, gene editing, and regenerative approaches are under investigation. Patient-physician "risk discussions" and tailored risk assessments are essential for improving patient outcomes.

In conclusion, the review underscores the complexity of the interconnected CKD and cardiovascular health domains. Ongoing research, innovative therapies, and personalized healthcare will be instrumental in addressing the challenges, reducing the disease burden, and enhancing well-being for individuals facing CKD and cardiovascular issues. Recognizing the intricate connections between these conditions is imperative for healthcare providers, policymakers, and researchers as they seek to improve the quality of care and outcomes for affected individuals.

## Introduction and background

Chronic Kidney Disease (CKD) has emerged as a progressively extensive worldwide healthcare subject [1]. Throughout its history, CKD has been described using various terms consisting of 'chronic renal failure,' 'pre-dialysis,' 'chronic renal insufficiency,' and 'pre-end-stage renal ailment', in large part depending on the underlying reasons [2]. An analysis of CKD necessitates the presence of kidney damage, confirmed through biopsy or damage markers, persisting for at least three months, both with or without a decline in glomerular filtration rate (GFR) or a GFR consistently below 60 mL/min per 1.73 m² for three months or longer, with or without concurrent kidney damage [3]. Any decrease in GFR shows functional kidney abnormalities, even though signs and symptoms like hematuria, albuminuria, unusual urinary sediment, or pathological kidney biopsy findings suggest structural abnormalities. These criteria should last at least three months to establish a CKD prognosis [4].

CKD often coexists with cardiovascular disease (CVD). The groundbreaking work of British physician Richard Bright illuminated the connection between CKD and CVD [5]. Individuals experiencing CKD face a great risk of cardiovascular events, with 50% of the patients in stages four to five experiencing CVD. Cardiovascular-associated mortality constitutes approximately forty to fifty percent of all deaths in advanced CKD (stage 4) and end-stage kidney disease (stage 5) patients. This compares to the twenty-six percent mortality observed in people with normal kidney function. This elevated danger spans beyond atherosclerosis-related issues like myocardial infarction and stroke; it also comprises coronary heart failure and fatal arrhythmias, specifically in advanced stages of CKD [6]. Even after adjusting for conventional cardiovascular risk factors, including high blood pressure, diabetes, and dyslipidemia, the effect of CKD on cardiovascular chance remains evident [7]. In earlier CKD stages, patients are susceptible to both fatal and non-fatal cardiovascular events [8]. It was observed in a recent cohort study that patients with early-stage CKD, even in the absence of vascular disorder clinical symptoms, carry an excess risk of subsequent coronary coronary heart disease [9]. So, we now recognize that CKD has become a distinct risk factor for CVD, making it just as significant as Coronary Artery Disease (CAD) when it comes to overall survival. It's important to note that for patients with CKD, the chances of mortality from cardiovascular events are higher than the likelihood of progressing to the point where kidney failure requires renal replacement therapy [4]. Indeed, we now acknowledge that CKD has emerged as an independent risk factor for CVD, holding a comparable significance to CAD in terms of overall survival. Notably, in individuals with CKD, the probability of mortality from cardiovascular events exceeds the likelihood of progressing to the stage where kidney failure necessitates renal replacement therapy [10].

This narrative review aims to comprehensively address the intricate relationship between these two critical healthcare domains. It synthesizes existing knowledge and explores recent research findings to offer insights into preventing, diagnosing, and managing cardiovascular complications in CKD patients. The scope of the review encompasses defining CKD stages, epidemiology, and risk factors, emphasizing the importance of cardiovascular health, elucidating pathophysiological mechanisms, detailing clinical manifestations, discussing diagnostic approaches, treatment options, challenges in managing both conditions, emerging therapies, and strategies to enhance patient outcomes. It provides a holistic perspective on this crucial intersection in healthcare.

## Review

Chronic kidney disease

CKD is a global healthcare challenge and affects 8% to 16% of the worldwide population. It often goes undetected by patients and healthcare providers. The definition of CKD comprises specific criteria, including a glomerular filtration rate (GFR) below 60 mL/min/1.73 m2, albuminuria of at least 30 mg per 24 hours, or evidence of kidney damage, such as hematuria or structural abnormalities, persisting for over 3 months [11]. Within the general population of the United States, the average annual decline in GFR is approximately 1 mL/min/1.73 m2, with a lifetime risk of GFR below 60 mL/min/1.73 m2 exceeding 50% [12-14].

CKD classification comprises two main parameters: glomerular filtration rate (GFR) and albuminuria levels [15]. The 2012 KDIGO CKD classification categorizes CKD into six stages (G1 to G5), with further subdivision of G3 into 3a and 3b. Additionally, it incorporates three grades of albuminuria (A1, A2, and A3), classified based on the urinary albumin-creatinine ratio [16]. The stages are defined in the following table (Table 1).

**Table 1:** Kidney Disease: Improving Global Outcomes (KIDGO) classification for chronic kidney disease GFR= glomerular filtration rate, ACR= Albumin-creatinine ratio

GFR classification	
G1	GFR ≥ 90 mL/min/1.73 m^2^
G2	GFR 60-89 mL/min/1.73 m^2^
G3a	GFR 45-59 mL/min/1.73 m^2^
G3b	GFR 30-44 mL/min/1.73 m^2^
G4	GFR 15-29 mL/min/1.73 m^2^
G5	GFR 30-44 mL/min/1.73 m^2^
Albuminuria	
A1	ACR < 30 mg/gm
A2	ACR 30-299 mg/gm
A3	ACR > 300 mg/gm

The risk of developing CKD is influenced by a number of variables, including genetic characteristics, family history, race, age, and gender. Low birth weight, African-American ancestry, getting older, or having renal disease in the family are a few major risk factors. The likelihood of getting CKD is also considerably increased by smoking, diabetes, obesity, and high blood pressure. Conditions like diabetes and hypertension can swiftly advance to end-stage renal disease if they are not appropriately controlled. Excessive alcohol use, exposure to heavy metals, high cholesterol, metabolic syndrome, the use of painkillers, a history of heart disease, cancer, hepatitis C infection, and HIV are additional risk factors [17].

An extensive epidemiological study, pooling data from 33 population-based studies, revealed that the global prevalence of CKD stages 1-5 was 10.4% in men and 11.8% in women aged ≥20 years. The age-standardized global prevalence of CKD stages 3-5 in adults aged ≥20 years was 4.7% in men and 5.8% in women, with prevalence rates varying by geography and income levels [18]. Over time, the prevalence of CKD has exhibited diverse trends. In the United States, the prevalence of CKD stages 1-4 increased from 11.8% in 1988-1994 to 14.2% in 2015-2016, although not in a linear fashion. In contrast, the prevalence of CKD stages 3-4 remained relatively stable [19]. A recent comprehensive systematic review and meta-analysis involving nearly 7 million patients found a global prevalence of 13.4% for CKD stages 1-5 and 10.6% for CKD stages 3-5 [20].

Cardiovascular health

CVD covers a wide range of heart and blood vessel disorders, making it a prominent cause of death on a global scale, particularly in Western nations. CVD poses a significant challenge to public health and healthcare expenses [21]. The cardiovascular system, consisting of the heart and blood vessels, is vital for maintaining overall health [22]. This intricate system faces various challenges, including irregularities in the conduction system, endocarditis, and rheumatic heart disease. However, when discussing CVD or heart disease, the primary focus is on four main categories: Peripheral artery disease (PAD), aortic atherosclerosis, cerebrovascular disease, and CAD [23]. These conditions collectively present substantial challenges to healthcare systems worldwide.

Cardiometabolic, behavioral, environmental, and social factors underlie the ongoing and escalating burden of CVD on healthcare systems. CVDs are alarmingly prevalent, linked to poor survival rates, and are increasing globally. The total number of CVD cases nearly doubled from 271 million in 1990 to 523 million in 2019, accompanied by a corresponding rise in CVD-related deaths from 12.1 million to 18.6 million during the same period [24]. The shifting health burden during this epidemiological transition presents a distinct challenge [25]. Developed regions have entered the fourth stage, marked by decreased infection-related conditions like rheumatic heart disease [26].

Meanwhile, emerging economies are dealing with a rise in noncommunicable diseases like CVD due to changing lifestyles and increased life expectancies. These transitions are closely intertwined with social and economic developments, adding complexity to the landscape. Effectively addressing the treatment gaps for prevalent CVD risk factors, such as hypertension and elevated cholesterol levels, remains a substantial challenge [25].

The ramifications of CVD encompass a broader spectrum of health concerns. Health conditions such as heart failure, atrial fibrillation, and CKD are progressively assuming greater significance within the healthcare domain [27-29]. Additionally, it's noteworthy that the economic burden associated with CVD surpasses that of other ailments, including Alzheimer's and diabetes, particularly in terms of indirect costs [21]. The global impact of CVD is undeniably profound, as CVD is responsible for one in every three deaths in the United States and one in every four deaths in Europe [30]. Projections indicate that by 2035, clinical manifestations of CVD will be prevalent in over 45% of the U.S. population [23]. In the development and advancement of CVD, lifestyle factors like diet, physical activity, body weight, stress, alcohol intake, and tobacco use are central influencers.

Additionally, the coexistence of other medical conditions, particularly obesity, diabetes, hypertension, and high cholesterol levels, heightens the overall risk profile [31]. It is crucial to acknowledge the significant role of nutrition as a preventive measure in CVD and its potential to manage associated risk factors. Scientific research highlights the significance of dietary patterns in preventing CVD-related fatalities and controlling risk factors [32,33]. The primary objective of public health initiatives is to pinpoint specific nutrients, dietary patterns, or food choices that enhance CVD prevention [31].

The connection between CKD and CVD

The connection between CKD and CVD is intricate and has deep roots in medical history, dating back to the pioneering observations of British physician Richard Bright [5]. Individuals with CKD face an elevated vulnerability to cardiovascular events, manifested by a striking fifty percent prevalence of CVD in those within CKD stages 4 to 5 [34]. Cardiovascular mortality is of even greater significance since it is a predominant cause of death of around forty to fifty percent of all fatalities among individuals with advanced CKD (stage 4) and those with end-stage kidney disease (stage 5). This elevated cardiovascular mortality rate exceeds the 26% observed in individuals with healthy kidney function [35,36].

People with CKD face an elevated cardiovascular risk, marked by severe atherosclerosis-related complications like heart attacks and strokes, as well as heart failure and potentially life-threatening irregular heart rhythms, especially in advanced CKD stages. Even when we account for typical cardiovascular risk factors like high blood pressure, diabetes, and abnormal cholesterol levels, studies involving CKD patients not yet on dialysis consistently highlight the significant influence of CKD on cardiovascular risk [6]. CVD and CKD share several common risk factors, including age, diabetes, hypertension, abnormal cholesterol levels, smoking, family history, and male gender. Additionally, CKD patients face unconventional risk factors, such as harmful uremic substances and disturbances in mineral balance, which further increase the risk of CVD [4]. CKD in itself serves as an independent risk factor for CVDs [37]. The estimated glomerular filtration rate (eGFR) and the presence of albumin in the urine are separate indicators of cardiovascular outcomes. Even mild albuminuria, regardless of declining kidney function, can raise the risk of CVD by two to four times [38,39].

In the context of CKD, complex physiological mechanisms underlie the development of cardiovascular complications. These mechanisms involve structural changes in the heart muscle and blood vessels, leading to conditions like cardiomyopathy, atherosclerosis, stiffening of arteries, calcification, and subsequent clinical consequences. These outcomes encompass heart failure, ischemic heart disease, strokes, cardiovascular-related deaths, and the progression of kidney disease to End-Stage Renal Disease (ESRD) [40]. However, the association between CKD and cardiovascular health issues is characterized by a multifaceted interplay. Traditional risk factors interact with CKD-specific factors, including harmful uremic substances, oxidative stress, inflammation, endothelial dysfunction, and vascular calcification, collectively contributing to the complex relationship between CVD and CKD [41].

Integral to this narrative is the concept of Cardiorenal Syndrome (CRS), which characterizes the dynamic interaction between the heart and kidneys, presenting various subtypes based on the failing organ and temporal pattern. However, the pathophysiology of Cardiorenal Syndrome (CRS) lies far beyond these two organs, comprising a multitude of factors, including uremia, anemia, neurohormonal imbalances, inflammatory processes, endothelial dysfunction, vascular calcification, and atherosclerosis [42]. Atherosclerosis, characterized by persistent endothelial dysfunction-driven chronic inflammation, constitutes a fundamental link between CVD and CKD [43]. CKD strongly connects with CAD, sparking inflammation in the blood vessels. Increased levels of inflammatory markers like C-reactive protein (CRP) and interleukin-6 (IL-6) are closely linked to a higher risk of heart attacks. The intricacies of this cardiovascular conundrum are further exacerbated by endothelial dysfunction, with albuminuria and CKD intricately associated with impaired endothelial functionality. Uremic toxins like asymmetric dimethylarginine (ADMA) and indoxyl sulfate (IS) disrupt the synthesis of endothelial nitric oxide (NO), perpetuating endothelial dysfunction [41]. Vascular calcification emerges as a common accompaniment of CKD, impairing coronary flow reserve and elevating cardiac events [44-46]. Its influence extends beyond the coronary arteries, affecting heart valves and the heart structure, leading to left ventricular hypertrophy, diastolic dysfunction, and fibrosis [47]. Additionally, extracellular vesicles (EVs) have emerged as messengers between cells, transferring biologically active substances and influencing inflammation and blood clotting in CKD and CAD. MicroRNAs (miRNAs), tiny non-coding RNA molecules, play a crucial role in regulating genes after transcription, and changes in miRNA patterns have been detected in CKD patients [41].

Clinical manifestations of cardiovascular complications in CKD

As CKD advances, the connection between risk factors specific to kidney health and cardiovascular issues becomes increasingly evident, elevating the risk of CVD in CKD patients. Even when we consider the usual risk factors for heart problems, reduced kidney function and higher levels of albumin in the urine significantly worsen the vulnerability to CVD, amplifying the risk by two to four times in this population. Ironically, CVD is frequently undiagnosed and untreated in CKD patients, posing a significant healthcare dilemma [48].

CAD is a primary cardiovascular concern in CKD, and its prevalence steadily increases as the estimated glomerular filtration rate (eGFR) decreases [49]. Heart Failure (HF) is closely intertwined with CKD, with nearly half of HF patients also suffering from CKD. This dual diagnosis increases mortality and hospitalization rates, and the worsening of HF and mortality risk goes hand in hand with declining renal function, regardless of age, HF duration, or diabetes status [50,51]. The diagnostic picture is further complicated by overlapping symptoms, such as difficulty breathing and swelling, which are familiar to both HF and CKD, making distinctions difficult [52]. Arrhythmias, especially Atrial Fibrillation (AF), are another consequence of CKD, affecting a considerable portion of CKD and dialysis patients. The connection between AF and CKD is a two-way street, with AF affecting CKD and its advancement. Sudden Cardiac Death (SCD) is a major worry for CKD patients, especially those with End-Stage Kidney Disease (ESKD) [53]. Valvular Heart Disease (VHD) is a common issue in CKD, negatively impacting survival rates. Even mild cases of aortic stenosis (AS) or mitral regurgitation (MR) are associated with more than a 50% higher 5-year mortality rate compared to those without CKD [54]. Valvular calcification, which is more common in CKD patients, worsens as kidney function declines and independently predicts adverse cardiovascular outcomes. Additionally, VHD shares symptoms like difficulty breathing and fatigue with CKD, which can complicate diagnosis [53].

Left ventricular hypertrophy (LVH), a precursor to congestive heart failure (HF), has an inverse relationship with the severity of renal dysfunction. While patients with stage G2 CKD only have a slightly elevated prevalence of LVH, it soars to 70-80% among dialysis patients [55]. Among CKD patients in stages G2-5 with heart failure (HF), about 60% have preserved ejection fraction (EF), while 40% have reduced EF, and the risk of death in both types of HF is inversely related to eGFR [56]. In patients undergoing regular dialysis, preserved EF HF is more common (about 80%) than reduced EF HF, and both predict a high risk of death [57]. The risk extends to strokes, with eGFR and proteinuria showing stepwise associations with the risk of incident stroke. CKD and albuminuria increase the risk of aortic aneurysms and peripheral vascular disease across all CKD stages, with higher levels of albuminuria intensifying these risks [58]. Among chronic dialysis patients, peripheral vascular disease is a significant concern, with a substantial 77% increased risk of mortality [59].

Diagnostic and monitoring strategies

The diagnosis of CKD typically necessitates at least two abnormal readings separated by a >3-month interval, making a confirmatory test a theoretical necessity [60]. In clinical practice, serum creatinine is the preferred marker for estimating eGFR due to its widespread availability and established history. However, equations incorporating both serum cystatin and creatinine offer reduced false positives and slightly improved diagnostic accuracy for CKD compared to those relying solely on serum creatinine [61]. Alternative approaches involve employing dipstick urinalysis or evaluating the urine albumin-creatinine ratio (ACR). Dipstick urinalysis proves valuable in identifying severe albuminuria, exhibiting a high predictive value for clinically significant cases [62]. However, its sensitivity diminishes when detecting less severe albuminuria, prompting concerns regarding potential misclassification [63]. ACR assessment, unaffected by operator skills, presents its own set of challenges, particularly in individuals with varying muscle mass, which can result in either underestimation or overestimation of albuminuria levels [64]. 

Evaluating the risk of CVD in CKD patients mainly depends on two crucial factors: glomerular filtration rate (GFR) and albuminuria. Although there is a consistent link between these indicators and cardiovascular risk, their added value in predicting heart-related issues beyond traditional risk factors is still a matter of discussion [65]. Various guidelines present differing risk categorizations, underlining the importance of personalized risk assessments. Recent research, exemplified by the CKD Prognosis Consortium's meta-analysis, accentuates the robust associations of both eGFR and ACR with cardiovascular mortality and heart failure, highlighting their predictive capabilities [38].

In addition to these measures, other biomarkers such as cystatin C, β2-microglobulin (B2M), and β-trace protein (BTP) have demonstrated promise in refining risk assessment. Furthermore, assessments of subclinical atherosclerosis through methods like coronary artery calcium (CAC), cardiac troponins, and natriuretic peptides contribute valuable insights into cardiovascular risk [65]. Mineral metabolism disorders include disruptions in serum phosphorus, calcium, and magnesium and increased vascular calcification promoters. Inflammation and oxidative stress biomarkers are under investigation for predicting cardiovascular events in CKD patients. Moreover, newly emerging biomarkers, such as micro-ribonucleic acids (microRNAs), are becoming increasingly significant in CVD studies involving CKD patients [66].

Non-invasive imaging techniques, including echocardiography, play a critical role in assessing uremic cardiomyopathy (UC), a heart condition associated with CKD. Advanced methods like speckle tracking echocardiography and 3D imaging improve the precision of echocardiographic diagnostics. Cardiac magnetic resonance (CMR) excels at tissue characterization, detecting early-stage fibrosis and edema in CKD. Although less frequently used, computed tomography can reveal significant incidental findings, such as cardiac and vascular calcifications, which hold prognostic significance [67].

Management and treatment

Various therapeutic strategies have become crucial for improving patient outcomes in the intricate domain of CKD and cardiovascular health management. Foundational to this pursuit are lifestyle modifications, which constitute the fundamental underpinning. Patients are advised to adopt a holistic health strategy that includes maintaining a balanced diet, staying physically active, managing weight sensibly, quitting smoking, and consuming alcohol in moderation [68]. These lifestyle changes promote general health and wellness and are central in preventing and managing chronic conditions, encompassing both CKD and heart problems. The reduction of protein intake and adoption of diets with lower acid loads hold the potential to mitigate kidney injury. Sodium restriction is remarkably advised, especially for hypertensive patients or those grappling with fluid overload [11].

Preventing cardiovascular events in CKD patients necessitates early-stage intervention, particularly in the presence of albuminuria, which predicts CKD progression and elevates CVD risk even with normal renal function [69]. Multimodal intervention in type 2 diabetes patients with microalbuminuria involving rigorous glucose control, statins, antihypertensive agents, aspirin, and lifestyle changes reduced vascular complications, cardiovascular risk, and overall mortality when compared to standard treatments [10]. CKD patients often need medication dose adjustments due to their impaired kidney function. Effective diabetes management is essential, with a target hemoglobin A1c of around 7.0% recommended by most guidelines. Additionally, dosage modifications might be necessary for oral hypoglycemic agents [11].

The treatment of cardiovascular issues in CKD is intricate and necessitates an individualized strategy to address the elevated cardiovascular risks linked to this condition. It is crucial to managing traditional risk factors, such as lowering systolic blood pressure within the target range of 130 to 139 mm Hg and prioritizing renin-angiotensin-aldosterone inhibitors as the initial choice for blood pressure control [70]. Customized management of blood sugar levels is of paramount importance, with a particular focus on avoiding hypoglycemic episodes, as they are linked to increased mortality in CKD patients [6]. When addressing CAD in CKD, invasive procedures like coronary angiography and revascularization are considered, but their advantages must be carefully weighed against the risk of stroke and mortality [6,71]. It's important to note that CKD patients often receive fewer evidence-based treatments, which contributes to higher mortality rates [72]. In the context of heart failure (HF), the focus is on heart function, and recommendations include the use of angiotensin-converting enzyme (ACE) inhibitors and β-blockers. However, data on their effectiveness in advanced CKD stages are limited [6]. In cases of ventricular arrhythmias and sudden cardiac death, implantable cardioverter-defibrillators (ICDs) are suggested, though concerns about their efficacy and value in CKD patients have been raised due to increased mortality [6,73]. Valve disorders are frequently seen in CKD, and transcatheter aortic valve implantation is a promising option, though it may affect kidney function [6].

Challenges and future directions

The management of the co-occurrence of Chronic Kidney Disease and cardiovascular health presents substantial challenges, as evidenced by the elevated levels of symptom burden, hospitalization, and mortality experienced by individuals facing both conditions [74]. The lack of comprehensive knowledge and awareness regarding CKD management further complicates the situation, highlighting the necessity for educational interventions for patients and healthcare professionals [75]. Additionally, CKD is often overlooked compared to other chronic illnesses, like congestive heart failure, despite its significant prevalence and costs; a more precise distinction between early and late-stage CKD is essential to garner the recognition and funding it deserves from health authorities [76].

Recent advances in research and therapeutic approaches offer potential solutions to these challenges. Sodium-glucose cotransporter-2 (SGLT2) inhibitors, initially developed for diabetes, have revealed remarkable cardiovascular and renal protective properties, mitigating the risk of kidney replacement therapy and mortality, even in advanced CKD stages [6,77]. Novel medications like selective nonsteroidal mineralocorticoid receptor antagonists (MRAs), such as finerenone, introduce innovative strategies by effectively countering the adverse effects of an overactive aldosterone system [6]. Similarly, glucagon-like peptide-1 receptor (GLP-1R) agonists and dipeptidyl peptidase-4 (DPP-4) inhibitors demonstrate renoprotective effects, albeit with lesser potency compared to SGLT2 inhibitors [78].

Furthermore, there is a potential therapeutic role for hypoxia-inducible factor (HIF) prolyl hydroxylase (PH) enzyme inhibitors like roxadustat in treating renal anemia associated with CKD [79]. However, their long-term effects on cardiovascular and renal health necessitate further investigation [78]. Promising outcomes are also observed with selective endothelin receptor antagonists and biological agents targeting the immune system, as they can potentially preserve renal function [80,81]. Stem cell-based therapies, including induced pluripotent stem cells and mesenchymal stem/stromal cells, offer regenerative possibilities but require additional research to assess their long-term safety and effectiveness [78]. CRISPR gene editing, an advanced technology with potential for gene therapy, is under investigation for treating CKD, targeting immune-mediated kidney diseases, and altering relevant genes in donor kidneys to address organ shortages [82,83].

Strategies aimed at enhancing patient outcomes encompass a risk-centered approach to lipid management and aspirin therapy, with the potential for tailored blood pressure management based on individualized assessments of cardiovascular risk [65,84]. Emphasizing the importance of patient-physician "risk discussions," which involve shared decision-making while considering predicted risk, treatment benefits, and patient preferences, is crucial [85]. However, the existing data on these aspects in CKD patients still need to be expanded, necessitating further exploration. Furthermore, there is a need to broaden the scope of risk prediction beyond commonly studied outcomes, such as sudden cardiac death, atrial fibrillation, and peripheral artery disease, to encompass those specifically relevant to CKD patients. Addressing these subtypes requires dedicated investigations and the development of targeted preventive and therapeutic strategies [65].

## Conclusions

In conclusion, the intricate interplay between CKD and cardiovascular health highlights the complexity of these closely linked health domains. This review underscores the heightened cardiovascular risk, especially in advanced CKD stages, where cardiovascular events, spanning from coronary artery disease to arrhythmias, are the primary contributors to illness and mortality. Precise risk assessment and early intervention are paramount for this vulnerable group, involving using biomarkers, advanced imaging, and tailored medication strategies. The CKD and cardiovascular management field is evolving, with promising treatments like SGLT2 inhibitors, MRAs, and GLP-1R agonists. Lifestyle modifications and patient-centered care remain central. Challenges in awareness, recognition, and the necessity for comprehensive, interdisciplinary care persist. In the future, ongoing research, innovative therapies, and personalized healthcare will be vital to address these challenges, enhance well-being, and alleviate the disease burden in this population. Recognizing the intricate connections between CKD and cardiovascular health is imperative for healthcare providers, policymakers, and researchers.
